# *Salmonella enterica* in the Chicken: How it has Helped Our Understanding of Immunology in a Non-Biomedical Model Species

**DOI:** 10.3389/fimmu.2014.00482

**Published:** 2014-10-10

**Authors:** Paul Wigley

**Affiliations:** ^1^Department of Infection Biology, School of Veterinary Science and Institute for Infection and Global Health, University of Liverpool, Liverpool, UK

**Keywords:** *Salmonella*, chickens, innate immunity, adaptive immune responses, immune regulation, heterophils, toll-like receptors, mucosal immune system

## Abstract

*Salmonella* infection of the chicken is important both as a source of foodborne human salmonellosis and as a source of disease in the chicken itself. Vaccination and other control strategies require an understanding of the immune response and as such have been important in understanding both mucosal immunity and more generally the response to bacterial infection. In this review, we discuss the contribution the study of avian salmonellosis has made to understanding innate immunity including the function of phagocytic cells, pattern recognition receptors, and defensins. The mucosal response to *Salmonella* infection and its regulation and the contribution this makes in protection against infection and persistence within the gut and future directions in better understanding the role of T_H_17 and Tregs in this response. Finally, we discuss the role of the immune system and its modulation in persistent infection and infection of the reproductive tract. We also outline key areas of research required to fully understand the interaction between the chicken immune system and *Salmonella* and how infection is maintained in the absence of substantive gastrointestinal disease.

## Introduction

*Salmonella enterica* has a close relationship with the chicken, as poultry meat and eggs are regarded as the most important source of human foodborne infection (1). Furthermore, host-adapted serovars of *Salmonella* are important worldwide pathogens of the chicken causing the fowl typhoid and pullorum disease (2). As a consequence, *S. enterica* is the most studied bacterial pathogen in the chicken, not as in the case of the mouse and other biomedical models to determine the mechanisms of infection and immunity related to human disease, but with a specific focus on its control in the poultry industry. As such the development of vaccines and potential immunotherapeutic agents and studies based on understanding the transmission and carriage of *Salmonella* have been critical to our understanding of the function of the avian immune system.

**Figure 1:**
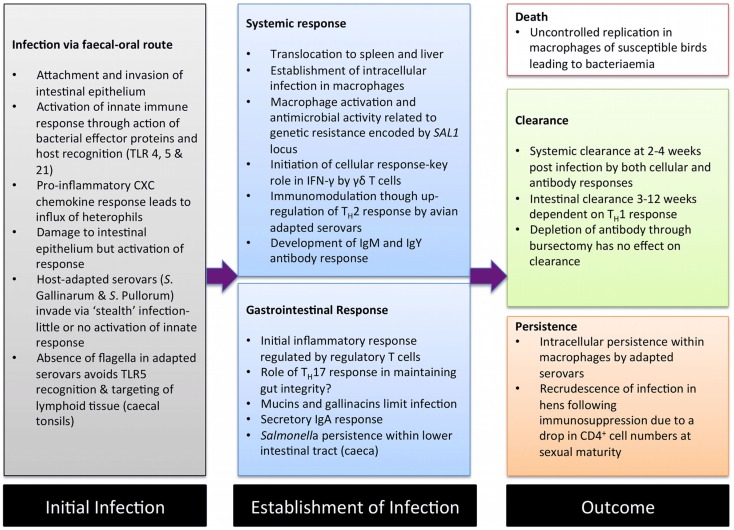
**A summary of the major interactions between *Salmonella enterica* and the chicken immune system**. During avian salmonellosis initial interactions between pathogen and host innate immunity occur in the intestinal epithelium. Progression of infection and the related immune response is related to the infecting serovar or strain and to the host-genetic background. *Salmonella* is frequently invasive in chickens leading to both systemic and mucosal responses. Typically, in resistant animals systemic infection is transient and cleared by the adaptive immune response. However, in susceptible animals where macrophages fail to limit infection, a disseminated infection resulting in death can occur. Clearance from the intestinal tract may take a number of months and is associated with cellular responses. Systemic persistence leading to a carrier state may occur, in particular with *S*. Pullorum with bacteria persisting in low numbers for the lifetime of the bird.

Avian salmonellosis can be broadly divided into two main types based on infection biology. The majority of broad-host range *S. enterica* serovars are capable of infecting the chicken, usually leading to a period of colonization of lower gastrointestinal tract. In some serovars, notably *S*. Typhimurium and *S*. Enteritidis, this may be accompanied by a low-level systemic infection that is resolved through cellular immunity within two-to-three weeks (3, 4). Colonization is usually accompanied by activation of inflammatory responses in the ileum and the two large blind caeca that branch off at the junction of the colon and ileum (5, 6). Although infection with these serovars can lead to systemic disease in chicks or immunocompromised animals, in healthy immunocompetent animals of a week of age or more, infection leads to little or no signs of disease. In contrast are the two adapted serovars *S*. Gallinarum, the cause of fowl typhoid, and *S*. Pullorum, the cause of Pullorum disease (2). These serovars lead to a systemic infection, often with high levels of morbidity and mortality (7) Unlike the broad-host range serovars invasion via the gut is not accompanied inflammation allowing the establishment of systemic infection while avoiding activation of immunity (6, 8, 9). This avoidance of innate activation has been termed “stealth infection” and is also employed by *Salmonella* Typhi in human beings (10). Colonization of the gut by avian-adapted serovars is also poor, largely as a consequence of “functional genomic shrinkage” with the loss of genes or accumulation of pseudogenes leading to a reduced metabolic capacity forcing them into a systemic intracellular lifestyle (11). As in mammalian models of infection, *Salmonella* invade and persist within macrophages and dendritic cells, and, as in mice, the progression of infection is to a large extent dependent on the susceptibility of the animal (9). In experimental fowl typhoid in a susceptible chicken, infection rapidly becomes disseminated leading to septicemia (5). In resistant animals, infection is better controlled by macrophages and eventually cleared via adaptive responses. *S*. Pullorum is generally a less virulent pathogen of the chicken, but can lead to a persistent systemic infection or carrier state that can in turn lead to infection of the mature reproductive tract of the hen (12). The stages of infection in avian salmonellosis and interactions with the immune systems are summarized in Figure 1.

The diversity of interactions with the host by *S. enterica* in the chicken, in both in terms of the tissues and cell types involved and the steps taken by the bacterium to avoid and manipulate the immune system has revealed many similarities between the mammalian and avian systems that broadly function in the same way when challenged by *Salmonella*, yet there are a number of, sometime subtle, differences that reflect 200 million years of divergent evolution.

## Major Differences between the Avian and Mammalian Immune Systems – A Brief Overview of a Compact Immune System

Functionally the immune system of the chicken behaves much the same way as that of mammals, perhaps reflecting a common ancestry. “Chickens are not feathered mice.” a comment made by Jim Kaufman, a leader in the field of avian immunogenetics, clearly illustrates that there are key structural and functional differences found between the classes. Generally, the chicken immune system is more compact, with less polymorphism in its receptors and all but the IL-15 multigene family having fewer members than its murine equivalent. This is perhaps most clearly illustrated by the MHC Class I of the chicken, which has only two alleles with one dominantly expressed, leading to it being termed the “minimal essential MHC” (13). The chicken has only three immunoglobulin classes IgG (or IgY), IgM, and IgA and no IgG subclasses. Although the chicken TCR is considered to be less polymorphic there are two variants of αβ T-cells termed TCR1 and TCR2 along with γδ cells, which, interestingly, are found in greater numbers in the chicken. Toll-like receptors also have the same broad structure and function as mammals and recognize a similar array of ligands, though differences are found perhaps most markedly the absence of TLR9, which is replaced functionally by TLR21 (14), and the presence of TLR15, which has no known equivalent in mammalian systems (15). A comprehensive description of the avian immune system can be found in the recently published 2nd edition of ‘Avian Immunology’ (16).

## Interaction with the Innate Immune System – Informing Phagocyte Function, Inflammation, and Toll-Like Receptors

*Salmonella* usually infects chickens via the fecal–oral route with spread from the intestinal tract primarily at the distal ileum and caeca of the bird (1). Invasion is an inflammatory process leading to expression of proinflammatory cytokines and the chemokines CXCLi1 and CXCLi2, considered the equivalent of mammalian IL-8 (5, 6, 17, 18). This in turn leads to an influx of heterophils and monocytic phagocytes to the gut resulting in inflammation and damage to the gut including fusion and flattening of the villi. Despite this enteropathogenic response, diarrheoa rarely occurs. While the bacterium itself induces cellular changes and inflammation through secreted effectors via its SPI1 Type III secretion system, recognition of flagellin through TLR5 appears to be the key event in the process (19). This is well illustrated by the fact that the non-flagellate avian-adapted serovars cause little inflammation during epithelial invasion *in vitro* or *in vivo* (9, 20), and that mutations in the flagellin gene of *Salmonella* Typhimurium lead to a more rapid invasion with lower initial levels of inflammatory signal (9, 19, 20). Indeed, this may be an evolutionary feature of adaptation to the avian host.

The consequence of activation of innate immunity is an influx of heterophils, the avian polymorphonuclear cell, and macrophages to the intestine. While these can lead to inflammatory damage, they also largely limit invasive disease. Our understanding of the biology and function of heterophils is almost entirely based on *Salmonella* infection studies. Depletion of heterophils changes *S*. Enteritidis from a gastrointestinal infection to a systemic infection illustrating their critical role in early immunity (21). Heterophils possess an array of TLRs (22), are efficient phagocytes, and can produce extracellular traps to facilitate this process (23). Unlike mammalian neutrophils, heterophils rely more on antimicrobial peptides as a bacterial killing mechanism (24) and although they produce nitric oxide and oxidative responses to *Salmonella* they lack the myeloperoxidase pathway (25). The study of the interaction of *Salmonella* with primary cultures of heterophils along with primary and continuous macrophage lines has been critical in our understanding of pattern recognition receptors in the chicken, including TLR5 as described above. Perhaps this is most clearly seen for TLR4 where variation in macrophage responses to *S*. Typhimurium challenge has identified both differences in levels of TLR4 expression and polymorphism in the receptor sequences between chicken lines. This suggests that responsiveness to LPS in chicken, which is frequently much lower than in mammals, is governed by variation in both levels of expression of the receptor and in the structure of the receptor itself (26, 27). Chicken TLR21 has no mammalian equivalent, though functionally it mirrors mammalian TLR9 in recognition of unmethylated (or CpG) DNA sequences. Much of our understanding of the response to CpG motifs has come through attempts to develop these sequences as immunostimulatory molecules or as vaccine adjuvant components to help control *Salmonella* (28, 29), although identification of the role of TLR21 was also founded in understanding the response to *Campylobacter jejuni* (14).

Macrophages differ little in structure or function to mammals, displaying a range of TLRs, expression of MHC Class II and phagocytic and antimicrobial activity. It is not yet understood whether avian macrophages have M1 or M2 phenotypes. The interaction with macrophages and dendritic cells and *Salmonella* is a key stage in the progression of systemic infection in particular. We have previously reviewed this in some detail (9), so will only briefly cover the essential points here. The use of inbred chicken models has identified the genetic locus *SAL1* that displays a phenotype of resistance to systemic salmonellosis (30). Macrophages derived from such birds shown enhanced oxidative killing and more rapid expression of key inflammatory and T_H_1-assocated cytokines (31, 32). Fine mapping of this resistance locus has identified Akt1, a protein kinase, and Siva, a CD27-binding protein as functional candidates for the *SAL1* locus (33). A number of chicken macrophage-like cell lines are available and these have been utilized extensively to understand the interactions between *Salmonella* and this cell type in terms of cytokine response, the role of the bacterial SPI2 type III secretion system in intracellular survival and antimicrobial response to a range of serovars and have largely shown a common biology between mammalian and avian species (34–40).

As mentioned previously antimicrobial peptides play a key role in protection against avian salmonellosis. β-Defensins termed gallinacins in the chicken are produced by a range of cells and tissues in response to *Salmonella* infection or vaccination including, but not restricted to gallinacins 2–5 and 7 in gut epithelium (41–43). Gallinacins are also expressed during reproductive tract infection as described below. Like their mammalian equivalents gallinacins are cysteine-rich antimicrobials that have been shown to be active against a range of Gram negative and Gram positive bacterial species and have been considered as potential therapeutics in human medicine (44). Cathelicidins, also termed fowlcidins in the chicken, have also been described, but their role in salmonellosis is not known (44, 45). Other innate factors including increased expression of mucins, and in particular the gel-forming mucins (Muc2, Muc5ac, Muc5b, and Muc6), are likely to play a role in maintaining the epithelial barrier and limiting infection. Purified chicken mucin has been shown to have activity against *Campylobacter* (46), and work is ongoing in out laboratory to determine its role in enteric infections.

## The Adaptive Response to Infection and the Success of Vaccination

The success of vaccination programs such as those employed in the UK to reduce the burden of foodborne salmonellosis through control in egg and latterly poultry meat production is a clear indicator that protective adaptive immune responses can be elicited in the chicken (47). Infection with *Salmonella* elicits both antibody and cellular responses that can be detected from around a week post-infection. Clearance of both *S*. Enteritidis and the attenuated *S*. Gallinarum 9R vaccine strain from the spleen and liver is at around 2–3 weeks post-infection which coincides with high levels of interferon-γ expression and also production of IgM and IgG antibodies (5, 7, 48, 49). Preliminary adoptive transfer experiments have shown partial protection to systemic infection can be achieved by transfer of T lymphocytes (9).

In contrast, clearance from the intestinal tract is a much slower process. *Salmonella* infection leads to production of secretory IgA in the gut but any protective role is unclear as studies employing bursectomised (B lymphocyte-free) chickens give differing results dependent on the method employed. Both clearance and protection to re-challenge with *Salmonella* are reduced when hormonal or cyclophosphamide are used to deplete the Bursa of Fabricius (50, 51), whereas surgical bursectomy *in ovo* has no effect on the clearance of *Salmonella* or protection to re-challenge (52). Whilst the latter study suggests antibody is not required for clearance, the success of inactivated vaccines in *Salmonella* control in the chicken does suggest it plays an important role. However a number of studies have shown that challenge elicits a strong Th1 response and that cellular immunity is more important in the chicken and clearance is dependent on age and cellular development. What we do not yet know is which effector mechanisms are employed in clearance. We do have some understanding of how the cellular response is activated. γδ-T lymphocytes are found in greater numbers in the chicken gut than mammalian systems and these cells play a key role in activation of adaptive response in the caeca and ileum. *Salmonella* challenge results in an influx of γδ lymphocytes and expression of IFN-γ, IL-12, and IL-18 leading to activation of T_H_1 responses (53, 54). The γδ lymphocyte population has a heterogenous structure and phenotype in the chicken, with association of subsets with particular tissues (55). In the caeca, the CD8^+^αα^+^ γδ population is thought to be the main activator of the adaptive response (56).

## Mucosal Responses and the Role of and Tregs and T_H_17 Cells

Given the importance of T_H_17 cells in the mucosal inflammatory response, and as sentinels in the intestinal epithelium in mammals, there has been little focus on their role in avian salmonellosis. Furthermore, our understanding of the regulation of inflammatory responses and the role of regulatory T-cells in maintaining gut integrity following inflammatory responses is also limited. T_H_17 cytokines are elicited rapidly after infection in the bovine ligated ileal loop *Salmonella* infection model (57), probably through stimulation of non-specific T_H_17 cells while *Salmonella*-specific T_H_17 cells possibly recognizing flagellin following activation via TLR5-dependent pathways may also contribute to intestinal mucosal protection (58). In the chicken IL-17 expression is upregulated in the cecum, the main site of bacterial colonization, following *S*. Enteritidis challenge though as yet no functional rule has been ascribed (42). Currently, the role of IL-17 is best characterized during infection by species of the chicken intestinal apicomplexan protozoan *Eimeria* where IL-17 may play a role both in protection and pathology dependent on the *Eimeria* species and co-infection with other enteric pathogens such as *Clostridium perfringens* (59–62).

The fact that many *Salmonella* serovars persist within the chicken intestinal tract with little sign of gastrointestinal disease despite eliciting a considerable inflammatory response and that inflammatory responses to *Salmonella* are relatively short-lived (5) strongly suggests there is a degree of regulation of this response. Our recent work on invasive *Salmonella* Typhimurium ST313 in the chicken illustrates this clearly (63); there is an initial CXCLi1 and CXCLi2 response leading to intestinal damage at three days post-oral infection, but by seven days post-infection this response is lowered and inflammatory damage largely resolved despite bacterial persistence (63). Some years ago, we showed that the lowering of intestinal proinflammatory signals following colonization with *S*. Typhimurium corresponded to increased expression of TGF-β, suggesting that regulation of inflammation was taking place (5). More recently the expression of IL-10 has been shown in the cecal tonsils in birds infected with *S*. Enteritidis at 4 days post-infection but not following infection the non-inflammatory avian-adapted serovars. It would seem likely that regulation of inflammatory immune responses, presumably by regulatory T-cells, allow *Salmonella* to persist within the gut for a number of weeks without disease to the bird but that the initial inflammatory response is sufficient to help control invasion and elicit responses that lead to systemic and eventually clearance of gastrointestinal infection. Such a “tolerogenic” response would have little or no impact on the bird itself, but has public health consequences in allowing persistence for several weeks, particularly given broiler chickens are typically slaughtered at around 6 weeks of age.

Recently, CD4^+^CD25^+^ cells have been identified as the avian equivalent of the mammalian Tregs, though the chicken appears to lack an ortholog of FoxP3 that are a characteristic feature of mammalian Tregs. Chicken CD4^+^CD25^+^ cells produce both IL-10 and TGF-β family cytokines and suppress T-cell proliferation *in vitro*. Stimulation of CD4^+^CD25^+^ *in vitro* or *in vivo* with *Salmonella* LPS, or infection, increases suppressive active. Intriguingly, CD4^+^CD25^+^ have also been shown to traffic to the cecal tonsil, suggesting this lymphoid organ at the ileal–cecal junction may play a key role in regulating intestinal immunity. There is clearly considerable scope to improve our understanding of chicken Tregs including the interaction with the intestinal microbiota, enteric pathogens, and in homeostasis of the healthy gut. Therapeutic approaches to deplete Treg function and thereby reduce suppression of the response to *Salmonella* have been proposed to reduce the carriage of *Salmonella* or *Campylobacter*. However, such an approach may well be detrimental to the health and welfare of chickens, leading to dysregulation of regulation of responses to the intestinal microflora resulting in poor gut health. Such an approach could also lead to uncontrolled inflammatory responses to *Salmonella* or *Campylobacter* infection leading to intestinal damage and diarrhea.

## Immunomodulation in Persistent Infections

A feature of avian salmonellosis is persistent infection or carrier state. Intestinal carriage may occur for several months following infection with broad-host range serovars such as *S*. Typhimurium and *S*. Enteritidis, whereas avian-adapted serovars, most notably *S*. Pullorum, may persist in low numbers within macrophages in the liver and spleen for the lifetime of the animal. This persistence is in the face of a substantial immune response requiring evasion or modulation of the response by the bacterium. As discussed above immune clearance in the chicken is likely to be centered on T_H_1-based cellular responses so avoiding these responses is key to pathogen survival. *S*. Pullorum is protected from antibody responses due to its intracellular niche, yet infection is associated with production of high titer IgG responses (12). Using a comparative approach between *S*. Pullorum and its close relative *S*. Enteritidis, we were able to show that systemic clearance of the latter was associated with a cellular response (9). In contrast, *S*. Pullorum infection leads to increased expression of IL-4 but unlike *S*. Enteritidis little expression of IFN-γ. This bias toward a T_H_2 response would allow *S*. Pullorum to establish an intracellular carrier state avoiding T_H_1-mediated clearance.

The mechanisms that underlie persistence in the GI tract are harder to determine. While as discussed above, regulation of the inflammatory response may help the establishment of a persistent infection, there is usually immune clearance in the long term. As with systemic infection, the level and length of intestinal colonization is influenced by the generic background of the host. A recent study using inbred White Leghorn chickens of Line 6_1_ considered susceptible to *Salmonella* colonization and Line N considered resistant (4), used a genome-wide transcriptional approaches to look at variations in enterocyte gene expression in an established GI tract infection (64). Both lines showed evidence of down-regulation of T_H_1 responses, little evidence of stimulation of the T_H_17 pathway, and no difference in expression of regulatory cytokines including IL-10 and TGF-β. In contrast the 6_1_ susceptible line showed enhanced expression of key T_H_2 cytokines including IL-4 and IL-13. This supports the notion that immune clearance of avian salmonellosis in T_H_1 dominated and that T_H_2 responses are associated with carrier states. As indicated by the authors, this is parallel with the murine model of *S*. Typhimurium where persistence is favored in M2 macrophage phenotypes that are driven by T_H_2 cytokine responses.

## Infection and the Immune Response in the Reproductive Tract

A unique feature of avian salmonellosis is the frequent infection of the female reproductive tract and transmission to eggs by *S*. Enteritidis and *S*. Pullorum (12, 65). The structure and function of the immune system of the avian reproductive has been recently reviewed, reflecting the considerable progress in our understanding of its structure and function made in the last few years (66). Infection by *Salmonella* or stimulation with LPS results in a local innate response and in particular secretion of gallinacins throughout the reproductive tract, but in particular the lower part of the oviduct and uterus (67–69). There is also an organized T lymphocyte structure in the developing tract and IL-4 expressed within the tract that can lead to specific IgA responses. Sexual maturity in the hen has a profound effect on both systemic and local lymphocyte populations with a temporary fall in circulating T lymphocytes and particular CD4^+^ cells and a loss of lymphocytic structure in the reproductive tract (70). This results in increased susceptibility to *Salmonella* challenge and decreased efficacy of vaccination at the start of the egg-laying period.

## Conclusion and Future Directions

Avian immunology has advanced greatly in recent years with the advent of genomic and transcriptomic approaches overcoming many of the difficulties due to lack of reagents, transgenic animals, or differences in the immune system that prevent the use of techniques commonly used in human and murine immunology. As transgenic chickens are now becoming available, functional studies on knockout chickens will no doubt follow. Nowhere will these be more welcomed than in understanding mucosal immunity, the “business end” of the response to *Salmonella*. There are a number of key questions that still need to be fully answered:

1. What are the mechanisms that underlie persistence of *Salmonella* in the chicken gut?
2. What regulates the GI response to prevent excessive intestinal damage?
3. Which effector mechanisms are important in clearance?

In addition to these, there are a number of areas, not least the role of microbiota in the development and homeostasis of the chicken mucosal immune system that require much work to improve our understanding of fundamental processes and mechanisms. While the ultimate aim of the avian *Salmonella* immunologist is to develop and improve vaccination and other controls that reduce the burden of *Salmonella* in food production, a better understanding of how the chicken regulates its response is as important, as disruption of this may have implications for the health and welfare of the animal itself, something that is increasingly important to the consumer.

## Conflict of Interest Statement

The author declares that the research was conducted in the absence of any commercial or financial relationships that could be construed as a potential conflict of interest.

## References

[1] Barrow PA Jones MA Smith AL Wigley P The long view: *Salmonella* – the last forty years. Avian Pathol (2012) 41:413–20 10.1080/03079457.2012.718071 23025669

[2] Barrow PA Freitas Neto OC Pullorum disease and fowl typhoid – new thoughts on old diseases: a review. Avian Pathol (2011) 40:1–13 10.1080/03079457.2010.542575 21331943

[3] Beal RK Powers C Wigley P Barrow PA Smith AL Temporal dynamics of the cellular, humoral and cytokine responses in chickens during primary and secondary infection with *Salmonella enterica* serovar Typhimurium. Avian Pathol (2004) 33:25–33 10.1080/03079450310001636282 14681065

[4] Barrow PA Bumstead N Marston K Lovell MA Wigley P Faecal shedding and intestinal colonization of *Salmonella enterica* in in-bred chickens: the effect of host-genetic background. Epidemiol Infect (2004) 132:117–26 10.1017/S0950268803001274 14979597 PMC2870085

[5] Withanage GS Wigley P Kaiser P Mastroeni P Brooks H Powers C Cytokine and chemokine responses associated with clearance of a primary *Salmonella enterica* serovar Typhimurium infection in the chicken and in protective immunity to rechallenge. Infect Immun (2005) 73:5173–82 10.1128/IAI.73.8.5173-5182.2005 16041035 PMC1201213

[6] Setta AM Barrow PA Kaiser P Jones MA Early immune dynamics following infection with *Salmonella enterica* serovars Enteritidis, infantis, pullorum and Gallinarum: cytokine and chemokine gene expression profile and cellular changes of chicken cecal tonsils. Comp Immunol Microbiol Infect Dis (2012) 35:397–410 10.1016/j.cimid.2012.03.004 22512820

[7] Wigley P Hulme S Powers C Beal R Smith A Barrow P Oral infection with the *Salmonella enterica* serovar Gallinarum 9R attenuated live vaccine as a model to characterise immunity to fowl typhoid in the chicken. BMC Vet Res (2005) 1:2 10.1186/1746-6148-1-2 16221297 PMC1236940

[8] Henderson SC Bounous DI Lee MD Early events in the pathogenesis of avian salmonellosis. Infect Immun (1999) 67:3580–6 10377142 10.1128/iai.67.7.3580-3586.1999 PMC116547

[9] Chappell L Kaiser P Barrow P Jones MA Johnston C Wigley P The immunobiology of avian systemic salmonellosis. Vet Immunol Immunopathol (2009) 128:53–9 10.1016/j.vetimm.2008.10.295 19070366

[10] Merrell DS Falkow S Frontal and stealth attack strategies in microbial pathogenesis. Nature (2004) 430:250–6 10.1038/nature02760 15241423

[11] Thomson NR Clayton DJ Windhorst D Vernikos G Davidson S Churcher C Comparative genome analysis of *Salmonella enteritidis* PT4 and *Salmonella* Gallinarum 287/91 provides insights into evolutionary and host adaptation pathways. Genome Res (2008) 18:1624–37 10.1101/gr.077404.108 18583645 PMC2556274

[12] Wigley P Berchieri A Jr Page KL Smith AL Barrow PA *Salmonella enterica* serovar Pullorum persists in splenic macrophages and in the reproductive tract during persistent, disease-free carriage in chickens. Infect Immun (2001) 69:7873–9 10.1128/IAI.69.12.7873-7879.2001 11705970 PMC98884

[13] Kaufman J Milne S Gobel TW Walker BA Jacob JP Auffray C The chicken B locus is a minimal essential major histocompatibility complex. Nature (1999) 401:923–5 10.1038/44856 10553909

[14] Keestra AM de Zoete MR Bouwman LI van Putten JP Chicken TLR21 is an innate CpG DNA receptor distinct from mammalian TLR9. J Immunol (2010) 185:460–7 10.4049/jimmunol.0901921 20498358

[15] Nerren JR Swaggerty CL MacKinnon KM Genovese KJ He H Pevzner I Differential mRNA expression of the avian-specific toll-like receptor 15 between heterophils from *Salmonella*-susceptible and -resistant chickens. Immunogenetics (2009) 61:71–7 10.1007/s00251-008-0340-0 19002681

[16] Schat KA Kaiser P Kaspers B Avian Immunology. London: Elsevier (2013).

[17] Withanage GS Kaiser P Wigley P Powers C Mastroeni P Brooks H Rapid expression of chemokines and proinflammatory cytokines in newly hatched chickens infected with *Salmonella enterica* serovar Typhimurium. Infect Immun (2004) 72:2152–9 10.1128/IAI.72.4.2152-2159.2004 15039338 PMC375210

[18] Matulova M Varmuzova K Sisak F Havlickova H Babak V Stejskal K Chicken innate immune response to oral infection with *Salmonella enterica* serovar Enteritidis. Vet Res (2013) 44:37 10.1186/1297-9716-44-37 23687968 PMC3663788

[19] Iqbal M Philbin VJ Withanage GS Wigley P Beal RK Goodchild MJ Identification and functional characterization of chicken toll-like receptor 5 reveals a fundamental role in the biology of infection with *Salmonella enterica* serovar Typhimurium. Infect Immun (2005) 73:2344–50 10.1128/IAI.73.4.2344-2350.2005 15784580 PMC1087448

[20] Kaiser P Rothwell L Galyov EE Barrow PA Burnside J Wigley P Differential cytokine expression in avian cells in response to invasion by *Salmonella* typhimurium, *Salmonella* enteritidis and *Salmonella* Gallinarum. Microbiology (2000) 146(Pt 12):3217–26 10.1099/00221287-146-12-3217 11101679

[21] Kogut MH Tellez GI McGruder ED Hargis BM Williams JD Corrier DE Heterophils are decisive components in the early responses of chickens to *Salmonella enteritidis* infections. Microb Pathog (1994) 16:141–51 10.1006/mpat.1994.1015 8047002

[22] Kogut MH Chiang HI Swaggerty CL Pevzner IY Zhou H Gene expression analysis of toll-like receptor pathways in heterophils from genetic chicken lines that differ in their susceptibility to *Salmonella enteritidis*. Front Genet (2012) 3:121 10.3389/fgene.2012.00121 22783275 PMC3389315

[23] Chuammitri P Ostojic J Andreasen CB Redmond SB Lamont SJ Palic D Chicken heterophil extracellular traps (HETs): novel defense mechanism of chicken heterophils. Vet Immunol Immunopathol (2009) 129:126–31 10.1016/j.vetimm.2008.12.013 19178950

[24] Kannan L Liyanage R Lay JO Rath NC Evaluation of beta defensin 2 production by chicken heterophils using direct MALDI mass spectrometry. Mol Immunol (2009) 46:3151–6 10.1016/j.molimm.2009.07.005 19665233

[25] Maxwell MH Robertson GW The avian heterophil leucocyte: a review. Worlds Poult Sci J (1998) 54:155–78 10.1079/WPS19980012

[26] Higgs R Cormican P Cahalane S Allan B Lloyd AT Meade K Induction of a novel chicken toll-like receptor following *Salmonella enterica* serovar Typhimurium infection. Infect Immun (2006) 74:1692–8 10.1128/IAI.74.3.1692-1698.2006 16495540 PMC1418683

[27] He H Genovese KJ Nisbet DJ Kogut MH Profile of toll-like receptor expressions and induction of nitric oxide synthesis by toll-like receptor agonists in chicken monocytes. Mol Immunol (2006) 43:783–9 10.1016/j.molimm.2005.07.002 16098593

[28] He H Genovese KJ Swaggerty CL Nisbet DJ Kogut MH In vivo priming heterophil innate immune functions and increasing resistance to *Salmonella enteritidis* infection in neonatal chickens by immune stimulatory CpG oligodeoxynucleotides. Vet Immunol Immunopathol (2007) 117:275–83 10.1016/j.vetimm.2007.03.002 17434210

[29] Xie H Raybourne RB Babu US Lillehoj HS Heckert RA CpG-induced immunomodulation and intracellular bacterial killing in a chicken macrophage cell line. Dev Comp Immunol (2003) 27:823–34 10.1016/S0145-305X(03)00079-X 12818639

[30] Mariani P Barrow PA Cheng HH Groenen MM Negrini R Bumstead N Localization to chicken chromosome 5 of a novel locus determining salmonellosis resistance. Immunogenetics (2001) 53:786–91 10.1007/s00251-001-0387-7 11862411

[31] Wigley P Hulme S Rothwell L Bumstead N Kaiser P Barrow P Macrophages isolated from chickens genetically resistant or susceptible to systemic salmonellosis show magnitudinal and temporal differential expression of cytokines and chemokines following *Salmonella enterica* challenge. Infect Immun (2006) 74:1425–30 10.1128/IAI.74.2.1425-1430.2006 16428798 PMC1360331

[32] Wigley P Hulme SD Bumstead N Barrow PA In vivo and in vitro studies of genetic resistance to systemic salmonellosis in the chicken encoded by the SAL1 locus. Microbes Infect (2002) 4:1111–20 10.1016/S1286-4579(02)01635-0 12361910

[33] Fife MS Salmon N Hocking PM Kaiser P Fine mapping of the chicken salmonellosis resistance locus (SAL1). Anim Genet (2009) 40:871–7 10.1111/j.1365-2052.2009.01930.x 20597881

[34] Babu US Sommers K Harrison LM Balan KV Effects of fructooligosaccharide-inulin on *Salmonella*-killing and inflammatory gene expression in chicken macrophages. Vet Immunol Immunopathol (2012) 149:92–6 10.1016/j.vetimm.2012.05.003 22627194

[35] Babu US Gaines DW Lillehoj H Raybourne RB Differential reactive oxygen and nitrogen production and clearance of *Salmonella* serovars by chicken and mouse macrophages. Dev Comp Immunol (2006) 30:942–53 10.1016/j.dci.2005.12.001 16427126

[36] Kramer J Visscher AH Wagenaar JA Jeurissen SH Entry and survival of *Salmonella enterica* serotype Enteritidis PT4 in chicken macrophage and lymphocyte cell lines. Vet Microbiol (2003) 91:147–55 10.1016/S0378-1135(02)00304-8 12458164

[37] Setta A Barrow PA Kaiser P Jones MA Immune dynamics following infection of avian macrophages and epithelial cells with typhoidal and non-typhoidal *Salmonella enterica* serovars; bacterial invasion and persistence, nitric oxide and oxygen production, differential host gene expression, NF-kappaB signalling and cell cytotoxicity. Vet Immunol Immunopathol (2012) 146:212–24 10.1016/j.vetimm.2012.03.008 22475571

[38] Withanage GS Mastroeni P Brooks HJ Maskell DJ McConnell I Oxidative and nitrosative responses of the chicken macrophage cell line MQ-NCSU to experimental *Salmonella* infection. Br Poult Sci (2005) 46:261–7 10.1080/00071660500098608 16050178

[39] Jones MA Wigley P Page KL Hulme SD Barrow PA *Salmonella* eInterica serovar *Gallinarum* requires the *Salmonella* pathogenicity island 2 type III secretion system but not the *Salmonella* pathogenicity island 1 type III secretion system for virulence in chickens. Infect Immun (2001) 69:5471–6 10.1128/IAI.69.9.5471-5476.2001 11500419 PMC98659

[40] He H Genovese KJ Swaggerty CL Nisbet DJ Kogut MH A comparative study on invasion, survival, modulation of oxidative burst, and nitric oxide responses of macrophages (HD11), and systemic infection in chickens by prevalent poultry *Salmonella* serovars. Foodborne Pathog Dis (2012) 9:1104–10 10.1089/fpd.2012.1233 23067396

[41] Akbari MR Haghighi HR Chambers JR Sharif S Expression of antimicrobial peptide genes in chicken cecal tonsils after treatment with probiotics and challenge with *Salmonella*. Poult Sci (2008) 87:82–82

[42] Crhanova M Hradecka H Faldynova M Matulova M Havlickova H Sisak F Immune response of chicken gut to natural colonization by gut microflora and to *Salmonella enterica* serovar Enteritidis infection. Infect Immun (2011) 79:2755–63 10.1128/IAI.01375-10 21555397 PMC3191970

[43] Hasenstein JR Lamont SJ Chicken gallinacin gene cluster associated with *Salmonella* response in advanced intercross line. Avian Dis (2007) 51:561–7 10.1637/0005-2086(2007)51[561:CGGCAW]2.0.CO;2 17626484

[44] Zhang G Sunkara LT Avian antimicrobial host defense peptides: from biology to therapeutic applications. Pharmaceuticals (Basel) (2014) 7:220–47 10.3390/ph7030220 24583933 PMC3978490

[45] Bommineni YR Pham GH Sunkara LT Achanta M Zhang G Immune regulatory activities of fowlicidin-1, a cathelicidin host defense peptide. Mol Immunol (2014) 59:55–63 10.1016/j.molimm.2014.01.004 24491488

[46] Alemka A Whelan S Gough R Clyne M Gallagher ME Carrington SD Purified chicken intestinal mucin attenuates *Campylobacter jejuni* pathogenicity in vitro. J Med Microbiol (2010) 59:898–903 10.1099/jmm.0.019315-0 20466838

[47] O’Brien SJ The “decline and fall” of nontyphoidal salmonella in the United Kingdom. Clin Infect Dis (2013) 56:705–10 10.1093/cid/cis967 23166188 PMC3563394

[48] Babu U Dalloul RA Okamura M Lillehoj HS Xie H Raybourne RB *Salmonella* Enteritidis clearance and immune responses in chickens following *Salmonella* vaccination and challenge. Vet Immunol Immunopathol (2004) 101:251–7 10.1016/j.vetimm.2004.05.002 15350755

[49] Okamura M Lillehoj HS Raybourne RB Babu US Heckert RA Cell-mediated immune responses to a killed *Salmonella* Enteritidis vaccine: lymphocyte proliferation, T-cell changes and interleukin-6 (IL-6), IL-1, IL-2, and IFN-gamma production. Comp Immunol Microbiol Infect Dis (2004) 27:255–72 10.1016/j.cimid.2003.12.001 15178000

[50] Desmidt M Ducatelle R Mast J Goddeeris BM Kaspers B Haesebrouck F Role of the humoral immune system in *Salmonella* Enteritidis phage type four infection in chickens. Vet Immunol Immunopathol (1998) 63:355–67 10.1016/S0165-2427(98)00112-3 9656424

[51] Arnold JW Holt PS Response to *Salmonella* Enteritidis infection by the immunocompromised avian host. Poult Sci (1995) 74:656–65 10.3382/ps.0740656 7792237

[52] Beal RK Powers C Davison TF Barrow PA Smith AL Clearance of enteric *Salmonella enterica* serovar Typhimurium in chickens is independent of B-cell function. Infect Immun (2006) 74:1442–4 10.1128/IAI.74.2.1442-1444.2006 16428801 PMC1360334

[53] Berndt A Methner U Gamma/delta T cell response of chickens after oral administration of attenuated and non-attenuated *Salmonella* Typhimurium strains. Vet Immunol Immunopathol (2001) 78:143–61 10.1016/S0165-2427(00)00264-6 11182154

[54] Berndt A Pieper J Methner U Circulating y delta T cells in response to *Salmonella enterica* serovar Enteritidis exposure in chickens. Infect Immun (2006) 74:3967–78 10.1128/IAI.01128-05 16790770 PMC1489728

[55] Pieper J Methner U Berndt A Heterogeneity of avian gammadelta T cells. Vet Immunol Immunopathol (2008) 124:241–52 10.1016/j.vetimm.2008.03.008 18455805

[56] Pieper J Methner U Berndt A Characterization of avian gammadelta T-cell subsets after *Salmonella enterica* serovar Typhimurium infection of chicks. Infect Immun (2011) 79:822–9 10.1128/IAI.00788-10 21078853 PMC3028855

[57] Keestra AM Godinez I Xavier MN Winter MG Winter SE Tsolis RM Early MyD88-dependent induction of interleukin-17A expression during *Salmonella colitis*. Infect Immun (2011) 79:3131–40 10.1128/IAI.00018-11 21576324 PMC3147556

[58] McGeachy MJ McSorley SJ Microbial-induced Th17: superhero or supervillain? J Immunol (2012) 189:3285–91 10.4049/jimmunol.1201834 22997231 PMC3652671

[59] Zhang L Liu R Song M Hu Y Pan B Cai J *Eimeria tenella*: interleukin 17 contributes to host immunopathology in the gut during experimental infection. Exp Parasitol (2013) 133:121–30 10.1016/j.exppara.2012.11.009 23201216

[60] Kim WH Jeong J Park AR Yim D Kim YH Kim KD Chicken IL-17F: identification and comparative expression analysis in *Eimeria*-infected chickens. Dev Comp Immunol (2012) 38:401–9 10.1016/j.dci.2012.08.002 22922588

[61] Min W Kim WH Lillehoj EP Lillehoj HS Recent progress in host immunity to avian coccidiosis: IL-17 family cytokines as sentinels of the intestinal mucosa. Dev Comp Immunol (2013) 41:418–28 10.1016/j.dci.2013.04.003 23583525

[62] Del Cacho E Gallego M Lillehoj HS Quilez J Lillehoj EP Ramo A IL-17A regulates *Eimeria tenella* schizont maturation and migration in avian coccidiosis. Vet Res (2014) 45:25 10.1186/1297-9716-45-25 24571471 PMC3975951

[63] Parsons BN Humphrey S Salisbury AM Mikoleit J Hinton JC Gordon MA Invasive non-typhoidal *Salmonella* Typhimurium ST313 are not host-restricted and have an invasive phenotype in experimentally infected chickens. PLoS Negl Trop Dis (2013) 7:e2487 10.1371/journal.pntd.0002487 24130915 PMC3794976

[64] Chausse AM Grepinet O Bottreau E Robert V Hennequet-Antier C Lalmanach AC Susceptibility to *Salmonella* carrier-state: a possible Th2 response in susceptible chicks. Vet Immunol Immunopathol (2014) 159:16–28 10.1016/j.vetimm.2014.03.001 24694400

[65] Berchieri A Jr Wigley P Page K Murphy CK Barrow PA Further studies on vertical transmission and persistence of *Salmonella enterica* serovar Enteritidis phage type 4 in chickens. Avian Pathol (2001) 30:297–310 10.1080/03079450120066304 19184915

[66] Wigley P Schat KA Barrow P The avian reprodcutive immune system. In: Schat KA Kaiser P Kaspers B, editors. Avian Immunology. London: Elsevier (2013). p. 265–74

[67] Michailidis G Anastasiadou M Froment P Changes in the expression of toll-like receptors in response to lipopolysaccharide in chicken sertoli cells. Reprod Domest Anim (2012) 47:97–97 10.1530/REP-14-0064 24920664

[68] Michailidis G Avdi M Argiriou A Transcriptional profiling of antimicrobial peptides avian beta-defensins in the chicken ovary during sexual maturation and in response to *Salmonella* Enteritidis infection. Res Vet Sci (2012) 92:60–5 10.1016/j.rvsc.2010.10.010 21071048

[69] Anastasiadou M Avdi M Michailidis G Expression of avian beta-defensins during growth and in response to *Salmonella* infection in the chicken testis and epididymis. Reprod Domest Anim (2012) 47:73–73

[70] Johnston CE Hartley C Salisbury AM Wigley P Immunological changes at point-of-lay increase susceptibility to *Salmonella enterica* serovar Enteritidis infection in vaccinated chickens. PLoS One (2012) 7:e48195 10.1371/journal.pone.0048195 23133568 PMC3485033

